# Optimization of five qPCR protocols toward the detection and the quantification of antimicrobial resistance genes in environmental samples

**DOI:** 10.1016/j.mex.2021.101488

**Published:** 2021-08-12

**Authors:** Roberta Tolosi, Lisa Carraro, Andrea Laconi, Alessandra Piccirillo

**Affiliations:** Department of Comparative Biomedicine and Food Science, University of Padua, Viale dell'Università 16, Legnaro, Padua 35020, Italy

**Keywords:** Antimicrobial resistance genes, qPCR, SYBRGreen

## Abstract

Here, we describe the optimization and validation of five quantitative PCR (qPCR) assays by employing the SYBRGreen chemistry paired with melting curve analysis to detect and quantify clinically relevant antimicrobial resistance genes (ARGs) (i.e. *ermB, bla_CTXM1-like_, bla_CMY-2_, qnrA* and *qnrS*) from environmental samples (i.e. soil and manure)*.* These five protocols accurately detected and quantified the aforementioned ARGs in complex environmental matrices and represent useful tools for both diagnostic and monitoring activities of resistant bacteria and ARGs into the environment.

## Graphical abstract

**Figure:**
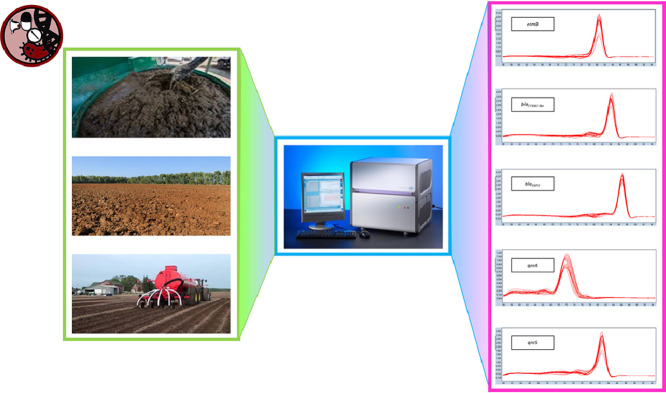

Specifications table

**Table:** 

Subject Area:	Immunology and Microbiology
More specific subject area:	Antimicrobial resistance genes detection and quantification
Protocol name:	SYBRgreen qPCRs paired with melting curve analysis for the detection and quantification of *ermB, bla_CTXM1-like_, bla_CMY-2_, qnrA* and *qnrS* genes in environmental samples.
Reagents/tools:	Reagents PowerUp™ SYBR® Green Master Mix (Thermo Fisher Scientific, USA) Primers (Macrogen, The Netherlands) ermB1 = 5’-CCGAACACTAGGGTTGCTC-3’ ermB2 = 5’-ATCTGGAACATCTGTGGTATG-3’ RTCTX-M-F = 5’-CTATGGCACCACCAACGATA-3’ RTCTX-M-R = 5’-ACGGCTTTCTGCCTTAGGTT-3’ FW3_CMY-2_Lahey = 5’-AGACGTTTAACGGCGTGTTG-3’ RV4_CMY-2_Lahey = 5’-TAAGTGCAGCAGGCGGATAC-3’ qnrAm-F = 5’-AGAGGATTTCTCACGCCAGG-3’ qnrAm-R = 5’-TGCCAGGCACAGATCTTGAC-3’ qnrSm-F = 5’-GCAAGTTCATTGAACAGGGT-3’ qnrSm-F = 5’-TCTAAACCGTCGAGTTCGGCG-3’
Experimental design:	This work represents the optimization of previously published PCR assays for the detection of five antimicrobial resistance genes (ARGs) by using the SYBRGreen chemistry paired with melting curve analysis. The primer pairs were tested firstly against DNA extracted from reference strains harbouring the target genes and then against total DNA extracted from complex environmental samples (i.e. soil and manure). These methods enable the detection and quantification of *ermB, bla_CTXM1-like_, bla_CMY-2_, qnrA* and *qnrS* genes.
Trial registration:	*NA*
Ethics:	*NA*
Value of the Protocol:	Rapid and reliable methods for the detection and quantification of clinically relevant antimicrobial resistance genes (ARGs) from complex matrices. Useful for research and monitoring of macrolide, (fluoro)quinolone and β-lactam resistance. Assays cheap and easy to adapt to the emergence of new ARG variants, being based on SYBRGreen chemistry.

## Description of protocol

The emergence of bacteria resistant to macrolides, (fluoro)quinolones and β-lactams represents a threat for human health, since antimicrobial drugs (AMDs) belonging to these classes are listed among the critically important antimicrobials by the World Health Organization (WHO) [1]. Of great concern is the dissemination in the environment of antimicrobial resistance genes (ARGs) conferring resistance to these AMDs, since they can be transferred to humans through different routes, including the food chain [2,3]. Here, we report the optimization of previously published assays, four end-point PCRs and one probe-based qPCR [4], [5], [6], [7], for the detection and quantification of five ARGs (i.e. *ermB, bla_CTXM1-like_, bla_CMY-2_, qnrA* and*, qnrS*) conferring resistance to the aforementioned AMDs classes. The SYBRGreen chemistry paired with the melting curve analysis was chosen being specific, cost-effective and easy to adapt to the emergence of new ARG variants. The optimization and then the validation of the assays were carried out by using DNA from both bacterial isolates and complex environmental samples (i.e. soil and manure). The analytical performances of the assays, i.e. specificity, dynamic range, limit of detection (LoD), limit of quantification (LoQ) and efficiency, were determined, and the amplicons from environmental samples were also analyzed by Sanger sequencing to further confirm the specificity of the assays.

## Major equipment and supplies for DNA extraction and quality/quantity assessment

Sterile 1.5 ml Eppendorf style microcentrifuge tubes (Sarstedt, Germany)

Adjustable micropipettes (0.5–1000 ml) (Gilson, USA)

Aerosol resistant micropipette tips (0.5–1000 ml) (Sarstedt, Germany)

Vortex Mixer (Velp, Italy)

Benchtop microcentrifuge (Eppendorf, Germany)

UV-Vis spectrophotometer NanoDrop ND-1000 (Nanodrop Technologies, USA).

Qubit 2.0 Fluorometer™ (Thermo Fisher Scientific, USA)

## Reagents for DNA extraction and quality/quantity assessment

Invisorb Spin Tissue Mini Kit (Invitek Molecular, Germany)

DNeasy PowerSoil kit (Qiagen, Germany)

Qubit™ dsDNA HS Assay Kit (Thermo Fisher Scientific, USA)

## Major equipment and supplies for qPCR and amplicon purification

LightCycler®480 Roche (Roche, Switzerland)

LightCycler®480 software version 1.5 (Roche, Switzerland)

Sterile 0.5–1.5–2.0 ml Eppendorf style microcentrifuge tubes (Sarstedt, Germany)

Adjustable micropipettes (0.1–1000 ml) (Gilson, USA)

Aerosol resistant micropipette tips (0.1–1000 ml) (Sarstedt, Germany)

Vortex Mixer (Velp, Italy)

Benchtop microcentrifuge 5424 (Eppendorf, Germany)

Plates centrifuge 5810R (Eppendorf, Germany)

Optically clear plates and foils for qPCR (Euroclone, Italy)

## Reagents for qPCR

PowerUp™ SYBR® Green Master Mix (Thermo Fisher Scientific, USA)

UltraPure™ DNase/RNase-Free Distilled Water (Thermo Fisher Scientific, USA)

Primer HPSF purified (Macrogen, The Netherlands). Primers stock concentration 100 μM

## Procedures

### DNA extraction

DNA extraction was performed under a sterile microbiological laminal flow cabinet to avoid contaminations. Micropipettes were used with aerosol resistant filter tips.

Genomic DNA was extracted from the reference strains (Table 1), kindly provided by the EU Reference Laboratory for Antimicrobial Resistance (DTU, Denmark), using the Invisorb Spin Tissue Mini Kit (Invitek Molecular, Germany) according to the manufacturer's instruction. DNeasy PowerSoil kit (Qiagen, Germany) was used to extract DNA from 93 environmental samples (i.e. soil and manure) following manufacturer's instructions. Both reference strains and environmental samples were stored at -80°C and thawed in ice.

**Table 1:** List of primers and reference strains.

Primer name	Sequence (5’–3’)	Annealing temperature	Amplicon size (bp)	Ref.	Reference strains
ermB1	CCGAACACTAGGGTTGCTC	56°C	139	[4]	*E. faecalis* JH2-2::Tn1545
ermB2	ATCTGGAACATCTGTGGTATG				
RTCTX-M-F	CTATGGCACCACCAACGATA	58°C	103	[7]	*E. coli* O 149 77-30108-11
RTCTX-M-R	ACGGCTTTCTGCCTTAGGTT				
FW3_CMY-2_Lahey	AGACGTTTAACGGCGTGTTG	58°C	128	[6]	*S. Heidelberg* 5-12893-1
RV4_CMY-2_Lahey	TAAGTGCAGCAGGCGGATAC				
qnrAm-F	AGAGGATTTCTCACGCCAGG	56°C	580	[5]	*E. cloacae* 03-577
qnrAm-R	TGCCAGGCACAGATCTTGAC				
qnrSm-F	GCAAGTTCATTGAACAGGGT	60°C	428	[5]	*E. coli* pHC19
qnrSm-F	TCTAAACCGTCGAGTTCGGCG				

DNA quantity was assessed by using the NanoDrop spectrophotometer (ThermoScientific, USA) and Qubit 2.0 Fluorometer (Thermo Fisher Scientific, USA), following the manufacturer's recommendations. A260/280 values equal or higher than 1.6 were considered acceptable. The extracted DNA was stored at -20°C until qPCR analysis.

### qPCR assays

The amplification mix was prepared under a sterile microbiological laminal flow cabinet to avoid contamination and all qPCR reagents were thawed in ice. Micropipettes were used with aerosol resistant filter tips. The cabinet and the pipettes were not the same used in DNA extraction steps. The qPCR assays were performed in a final volume of 10 μl reaction mixtures, containing 2.5 μl of DNA template, 1.3 μl of UltraPure™ DNase/RNase-Free Distilled Water (Thermo Fisher Scientific, USA), 5 μl of 2X PowerUp™ SYBR® Green Master Mix (Thermo Fisher Scientific, USA) and 0.6 μl of each primer (Table 1). The composition of the reagents in the qPCR mix is reported in the Table 2. qPCR mixes were mixed using the vortex and aliquot in optically clear plates kept in ice until being placed in the real-time machine. All qPCRs were performed in a LightCycler®480 Roche (Roche, Switzerland) real-time platform. All qPCR reactions were performed in triplicate.

**Table 2:** qPCR reaction mix.

Reagents	Final concentration	μl per reaction
PowerUp™ SYBR® Green Master Mix 2X	1X	5
Primer forward 10 μM	600 nM	0.6
Primer reverse 10 μM	600 nM	0.6
UltraPure™ DNase/RNase-Free Distilled Water	-	1.3
DNA template	-	2.5

The following PCR thermal profile was used: initial incubation at 50°C for 2 min, followed by 2 min at 95°C, and 45 cycles at 95°C for 10 s and 56–60°C for 40 s. The exact annealing temperatures of the assays are depicted in Table 1. Melting curves were determined by adding a dissociation step after the last amplification cycle with a temperature transition rate of 4.4°C/s between 40 and 95°C.

For the analysis of the environmental samples, in each plate a “positive control” (i.e. DNA obtained from the strain harbouring the ARG under analysis) was included, together with a “No Template Controls” (NTC) (i.e. a sample containing all qPCR reagents with the exception of the DNA template).

### Optimization of qPCR conditions

Assay optimization is crucial to ensuring the best qPCR performances. We optimized the annealing temperatures used in the previously published studies by comparing the amplification plots and dissociation curves. Furthermore, we tested different concentrations of each primer (300/300 nM, 300/600 nM, 600/300 nM, 600/600 nM, 600/900 nM, 900/600 nM and 900/900 nM for forward and reverse primer, respectively). The combination of concentrations yielding the lowest Cp, the best efficiency of amplification as well as negative NTC was chosen for the validation steps.

### Interpretation of qPCR amplification plots

**Fig. 1:**
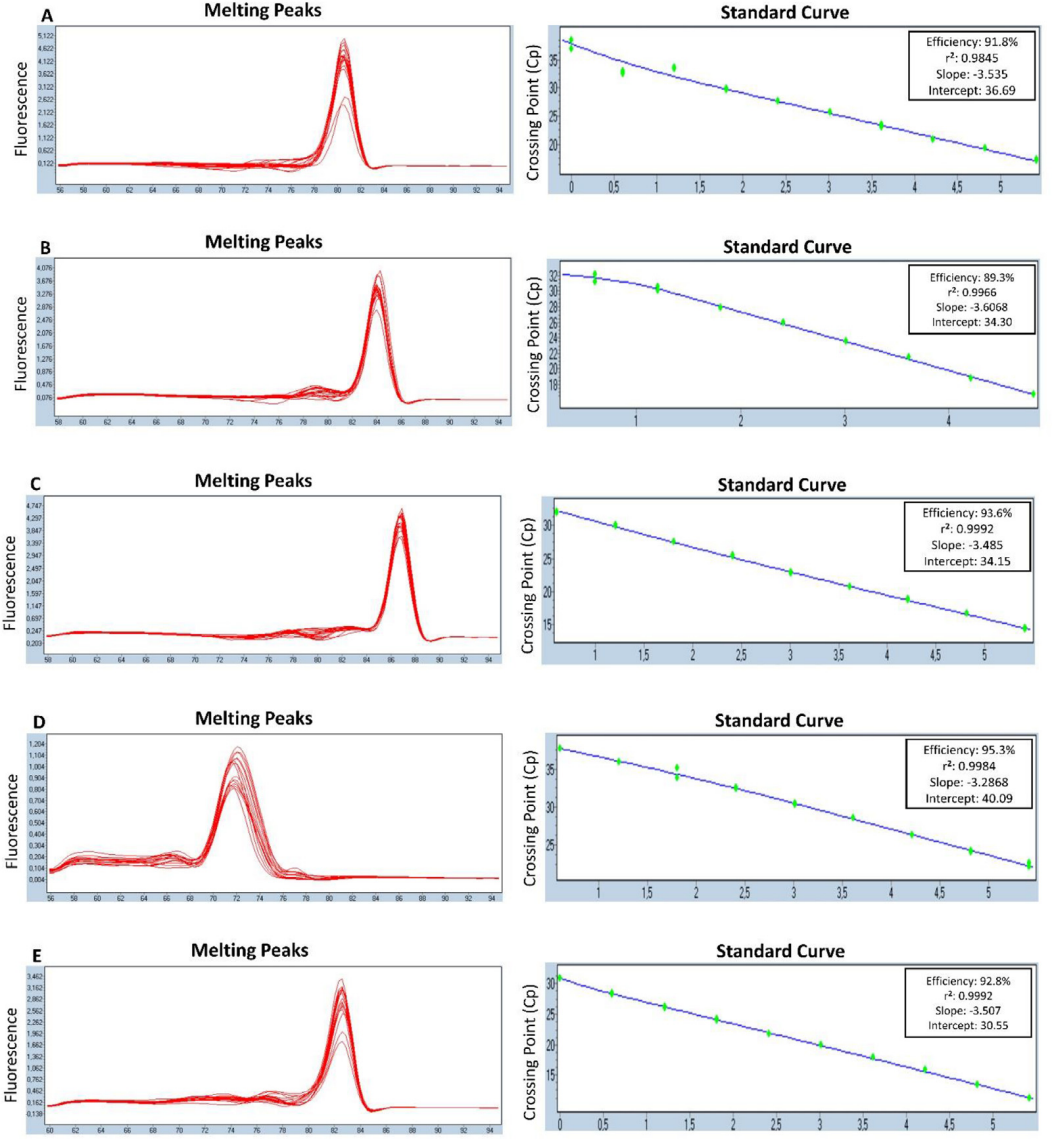
qPCR standard curves and melting curves for *ermB* (A)*, bla_CTXM1-like_* (B)*, bla_CMY-2_* (C)*, qnrA* (D) and*, qnrS* (E). For each standard curve, efficiency, slope, intercept, and r^2^ are reported.

**Fig. 2:**
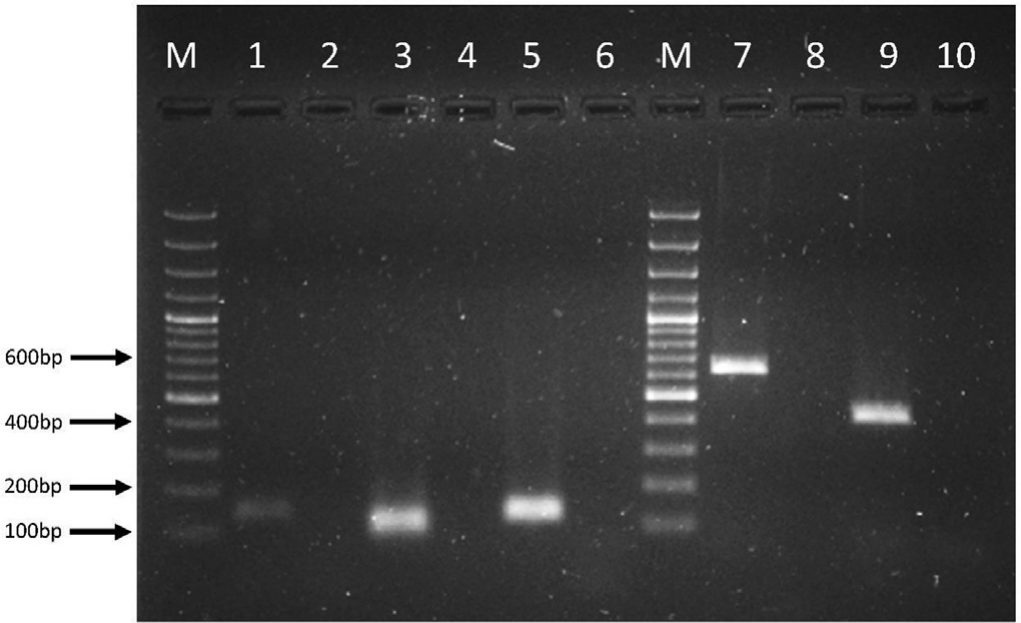
Agarose gel electrophoresis of amplicons obtained for each assay. Lane M = 100bp DNA ladder, lane 1 = *E. faecalis* JH2 2::Tn1545 (*ermB*), lane 2 = negative control *ermB*, lane 3 = *E. coli* O 149 77-30108-11 (*bla_CTXM1-like_*), lane 4 = negative control *bla_CTXM1-like_*, lane 5 = *S. Heidelberg* 5-12893-1 (*bla_CMY-2_*), lane 6 = negative control *bla_CMY-2_*, lane M = 100bp ladder, lane 7 = *E. cloacae* 03-577 (*qnrA*), lane 8 = negative control *qnrA*, lane 9 = *E. coli* pHC19 (*qnrS*), lane 10 = negative control *qnrS*.

For each run the baseline and the Crossing Point (Cp) of the amplification curves were calculated using the LightCycler®480 software version 1.5 (Roche). The specificity of the amplification was assessed by melting curve analysis. The melting temperature for *ermB, bla_CTXM1-like_, bla_CMY-2_, qnrA* and *qnrS* were 80.5 ± 0.2°C, 84.4 ± 0.2°C, 86.9 ± 0.2°C, 72.5 ± 0.2°C and 82.5 ± 0.2°C, respectively. As shown in Fig. 1, each of the optimized assays was able to identify correctly its target ARG based on the melting curve analysis. No amplification was observed for any NTCs. To confirm the specificity of the amplifications, PCR products of each assay were run on 1.5% gel; all amplicons showed the expected size (bp) and no non-specific bands were observed (Fig. 2).

### Analytical specificity

To avoid false positive results, each assay was tested against DNAs extracted from bacteria not harbouring its target gene. This experimental setting enabled to evaluate the cross-reactivity among the assays; no increase in fluorescence associated with a sigmoidal amplification curve was observed for any assays when tested against no-target genes (Supplementary material 1).

### Efficiency, analytical sensitivity and intra-assay variability

Positive control DNAs (previously amplified by end-point PCR) of known concentration (ng/μl) were serially diluted (1: 4 or 1: 5) and used to construct the standard curves. The Cp values of these standards were plotted against the logarithm of their concentrations. The technical assay dynamic range along with the limit of detection (LoD), the limit of quantification (LoQ), and the efficiency of the assays was determined. The correlation coefficient (r^2^), which provides an estimate of the goodness of fit of the data points to the linear trend-line, was also calculated. As depicted in Table 3, all assays show good efficiency (from 89.6 to 95.3%), linearity (r^2^ > 0.98), LoQ (from 8.13 to 320.80 gene copies number) and LoD (from 1.50 to 35.94 gene copies number). Furthermore, all assays showed good intra-assay repeatability, and mean Ct (three replicates), standard deviation (SD), and coefficient of variation (CV) for low (i.e. LoD), medium and high concentrations are reported in Table 4.

**Table 3:** Efficiency, r^2^, dynamic range, LoD and LoQ of the five assays.

Target gene	Efficiency	r^2^	Dynamic Range (Cp values)	LoD (copies number)	LoQ (copies number)
*ermB*	91.8%	0.9845	17.18-37.69	5.01	320.80
*bla_CTXM1-like_*	89.3%	0.9966	16.72-31.62	35.94	57.50
*bla_CMY-2_*	93.6%	0.9992	14.45-31.94	28.92	46.27
*qnrA*	95.3%	0.9984	22.32 - 37.77	1.50	96.10
*qnrS*	92,8%	0.9992	11.25-30.84	2.03	8.13

**Table 4:** Repeatability of the qPCR assays. Mean Ct (three replicates), standard deviation (SD) and coefficient of variation (CV) are reported for each ARG according to target gene DNA concentration.

Target gene	Concentration ng/μl	Mean	SD	CV
*ermB*	4.25E-05	19.26	±0.09	0.48
	6.60E-07	25.67	±0.03	0.11
	6.48E-10	37.69	±1.12	2.96
*bla_CTXM1-like_*	4.25E-05	16.72	±0.06	0.34
	6.60E-07	23.55	±0.07	0.30
	2.59E-09	31.62	±0.66	2.10
*bla_CMY-2_*	4.25E-05	16.67	±0.01	0.04
	6.64E-07	22.85	±0.07	0.31
	2.59E-09	31.93	±0.19	0.60
*qnrA*	4.25E-05	24.08	±0.20	0.82
	6.64E-07	30.44	±0.16	0.51
	2.59E-09	37.77	±0.85	2.25
*qnrS*	4.25E-05	13.37	±0.01	0.05
	6.64E-07	19.94	±0.02	0.11
	6.48E-10	30.84	±0.03	0.09

### Prevalence and absolute abundance of target ARGs in environmental samples

The assays were tested against 93 environmental samples (i.e. soil and manure); *ermB* was the gene showing the highest prevalence (81.72%), followed by *bla_CMY-2_* (58.06%), *bla_CTXM1-like_* (30.11%) and *qnrS* (24.73%), while *qnrA* was not detected in any sample. To further confirm the specificity of the assays, the amplicons obtained from the environmental samples were Sanger sequenced and BLAST searched against the Comprehensive Antibiotic Resistance Database (CARD, https://card.mcmaster.ca) using the FASTA sequences; 100% agreement between the results yielded by the qPCR assays and the sequences was observed. Kappa (ƙ) values were calculated as a measure of overall agreement between each qPCR assay and the sequencing results, which proved to be perfect (ƙ = 1). Statistical analysis was performed using in GraphPad Prism version 9.1.1 (https://www.graphpad.com). The absolute abundance of each ARG in the environmental samples was calculated based on the respective standard curve and it ranges from below the LoQ to 206.28, 242.38, 229.09 and 134.659 copies number for *ermB, bla_CTXM1-like_, bla_CMY-2_* and *qnrS,* respectively. However, the absolute abundance of ARGs in a given sample is not a significant value, as it is proportional to the total DNA present in the sample; therefore, 16S rRNA gene copy number should be obtained (e.g. by analysing the samples with the qPCR assay developed by Nadkarni et al. [8]) and ARGs relative abundance should be calculated by normalizing the ARG copy number to 16S rRNA gene copies.

## Declaration of Competing Interest

None.

## Appendix Supplementary materials

Image, application 1
