# Supplementary material for: Optimization of five qPCR protocols toward the detection and the quantification of antimicrobial resistance genes in environmental samples
Source: MethodsX. 2021 Aug 12;8:101488. doi: 10.1016/j.mex.2021.101488 (PMC8563462; doi:10.1016/j.mex.2021.101488)
Supplement: Supplementary file 1 [file mmc1.pptx]

## Slide 1
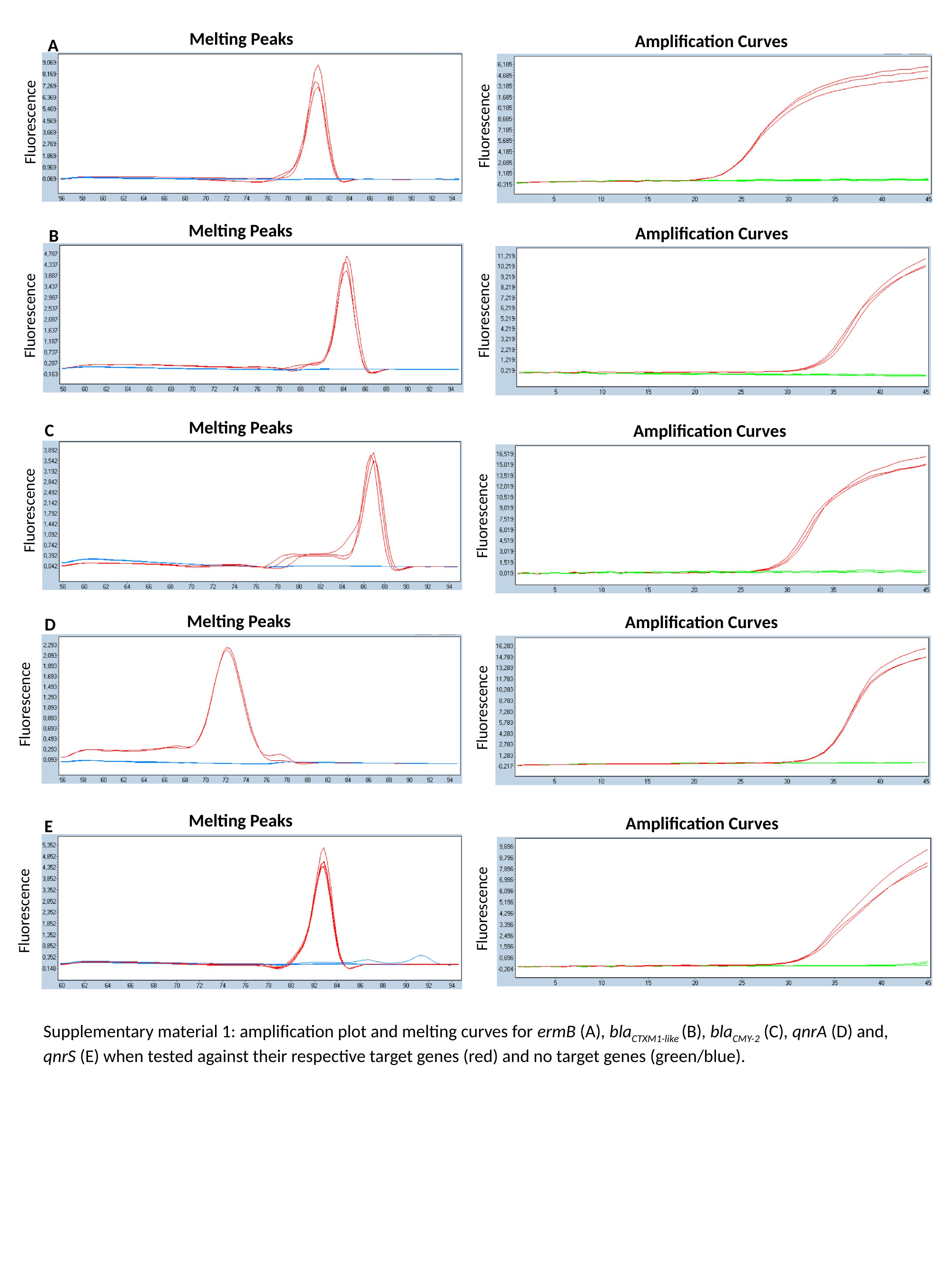

Melting Peaks
Amplification Curves
A
Fluorescence
Fluorescence
Melting Peaks
Amplification Curves
B
Fluorescence
Fluorescence
Melting Peaks
C
Amplification Curves
Fluorescence
Fluorescence
Melting Peaks
Amplification Curves
D
Fluorescence
Fluorescence
Melting Peaks
Amplification Curves
E
Fluorescence
Fluorescence
Supplementary material 1: amplification plot and melting curves for ermB (A), blaCTXM1-like (B), blaCMY-2 (C), qnrA (D) and, qnrS (E) when tested against their respective target genes (red) and no target genes (green/blue).
